# Hemorrhage after Extracting

**Published:** 1870-12

**Authors:** L. N. Hutchinson

**Affiliations:** Big Rapids, Mich.


### HEMORRHAGE AFTER EXTRACTING.

BY L. N. HUTCHINSON.

As the Dental Register and Cosmos are welcome visitors to my office, I read with much satisfaction all they contain; and although I have had the experience of twenty-five years in Dentistry, I never read them but I can learn something new. I do like to know how others work, and learn of their experience. It is a great aid to me, and I some times feel guilty to think I am always receiving and never contributing; then again I think my knowledge is so little it would not be acceptable. But as hemorrhage has baffled many *professional Dentists,* I would give my little experience. Some years ago I had a severe case. It continued nearly forty-eight hours. I had tried all remedies I was in possession of, tannin and subsulphate of iron with the rest. I was completely exhausted. Just at that time I came across a paper of *sugar lead.* Knowing it to be something of an astringent I mixed some dry with tannin on cotton and applied a compress, and to my great satisfaction it proved successful. Since that time I have had quite a number of cases, and it has never failed on the first application.

Big Rapids, Mich.

